# Acid-Based Deep Eutectic Solvents for Structural Modification of Sulphite Pulp Cellulose: A Potential Route Toward Advanced Materials

**DOI:** 10.3390/polym18131659

**Published:** 2026-07-03

**Authors:** María Guadalupe Morán-Aguilar, Iván Costa-Trigo, José Manuel Domínguez, Fabiola Vilaseca

**Affiliations:** 1 Advanced Biomaterials and Nanotechnology (BIMATEC), Department of Chemical and Agricultural Engineering, and Agrifood Technology, Polytechnic School, University of Girona, Maria Aurèlia Capmany 61, 17003 Girona, Spain; 2 LNEG—Laboratório Nacional de Energia e Geologia, Unidade de Bioenergia e Biorrefinarias, 1600-545 Lisboa, Portugal; ivan.trigo@lneg.pt; 3 Industrial Biotechnology and Environmental Engineering Group “BiotecnIA”, Chemical Engineering Department, University of Vigo (Campus Ourense), 32004 Ourense, Spain; jmanuel@uvigo.es

**Keywords:** deep eutectic solvents, cellulose modification, sulphite pulp pretreatment, sustainable cellulose processing, low-impact pretreatment, DES-modified sulphite pulp, biomaterial reinforcement

## Abstract

The transition toward renewable and environmentally responsible materials has intensified interest in cellulose-based systems for use in sustainable packaging applications. Although cellulose offers biocompatibility, structural versatility, and tuneable physicochemical properties, conventional modification routes rely on harsh chemicals and generate environmentally burdensome effluents. In this study, an efficient and a potentially green strategy for cellulose modification was developed using acid-based deep eutectic solvents (DES) composed of choline chloride and lactic, acetic, or citric acid at different molar ratios. Under mild conditions (110 °C, 4 h), DES pretreatment reduced glucan content in sulphite pulp from 99% to 79–93%, depending on the hydrogen bond donor (HBD), while suggesting an apparent increase in relative crystallinity, from approximately 82% to 90%, as estimated by the Segal method. FTIR, XRD, and morphological analyses revealed the disruption of the hydrogen bonding network, enhanced fibrillation, and residual DES-derived functional groups detectable by FTIR. Although DES pretreatment increased structural order, it also reduced enzymatic digestibility due to the higher proportion of crystalline domains. Overall, the results demonstrate that acidic DES constitutes a sustainable and recyclable medium capable of modulating cellulose structure and generating materials with enhanced physicochemical properties. These findings suggest that DES-modified cellulose could serve as a potential reinforcement platform for future biodegradable packaging and bioplastic formulations, enabling the development of high-performance, renewable, and environmentally compliant packaging materials.

## 1. Introduction

The use of renewable resources to develop environmentally friendly materials, while avoiding the use or generation of harmful substances, has become a leading international research frontier [1]. Numerous natural polymers—including polysaccharides, chitins, proteins, and various composite materials derived from renewable resources—have been extensively studied and applied as potential biomaterials and biodegradable packaging materials, owing to their unique structures and excellent functional properties that enable a wide range of applications [2,3].

Cellulose, the most abundant natural polymer, is a polysaccharide composed of a linear chain of several hundred to over 10,000 β (1→4)-linked D-glucose units [1]. It can be used to produce a wide range of renewable, biodegradable, biocompatible, and chemically derivable materials [4]. In this sense, cellulose has gained increasing attention due to its unique molecular architecture and advantageous physicochemical properties, which position it as an excellent raw material for the development of high-value-added functional biomaterials and biodegradable packaging materials [5]. Nevertheless, various physical, chemical, and biological strategies have been developed to enhance the reactivity of dissolving pulp, a key prerequisite for the efficient synthesis of cellulose derivatives [6].

Traditional strategies for producing cellulose derivatives—including mechanical, chemical, and enzymatic approaches—present important limitations. Mechanical treatments generally exhibit low efficiency and high energy demand, while conventional chemical processes such as acid hydrolysis and alkaline pretreatments (e.g., H_2_SO_4_, NaOH, KOH) rely on corrosive and environmentally hazardous reagents, generate effluents that are difficult to treat, require large volumes of water for pulp washing, and accelerate equipment corrosion [7,8,9]. Enzymatic pretreatments—particularly those based on endoglucanase—offer milder and more environmentally compatible conditions, yet their industrial deployment remains constrained by high costs and relatively low overall efficiencies [5,10,11]. These combined drawbacks have intensified the search for more sustainable, efficient, and economically viable alternatives for cellulose extraction, modification, and derivative production [12,13,14].

In response to these challenges, the pursuit of sustainable and efficient strategies for the extraction, modification, and functionalization of cellulose and its derivatives has positioned deep eutectic solvents (DES) as a promising alternative. DES constitutes an innovative class of green solvents capable of enhancing fibre swelling and macromolecular chain depolymerization through hydrogen-bond disruption [15]. Their low toxicity, biodegradability, tuneable physicochemical properties, and relatively simple preparation have further supported their use in cellulose pretreatment and processing [16].

The performance of DES-based pretreatments is strongly influenced by several parameters, including the DES composition—specifically the nature of the hydrogen-bond donor (HBD) and hydrogen-bond acceptor (HBA)—as well as the HBA: HBD molar ratio, temperature, pretreatment duration, and the intrinsic characteristics of the biomass source [17]. Recent studies have demonstrated that DES can improve cellulose accessibility, increase enzymatic hydrolysis yields, and facilitate the production of cellulose nanofibres, hydrogels, and other value-added biomaterials. Moreover, DES can be specifically formulated to exhibit tailored functionalities, enabling application-oriented and customizable cellulose processing [18].

Given their environmental compatibility and versatility, DES represent a sustainable platform for cellulose pretreatment across fields ranging from biorefinery to materials science [19]. A precise understanding of DES–cellulose interactions is critical, as pretreatment efficiency depends on both the chemical composition of the biomass and the specific formulation of the DES [20]. Particularly, it is necessary to determine how DES composition, molar ratio, and component-specific interactions govern fibre swelling, disruption of lignin–carbohydrate linkages, and the resulting increase in cellulose accessibility [21]. Establishing these structure–property relationships is essential for selecting or designing DES systems tailored to specific biomass types and for enabling the efficient production of cellulose-derived reinforcements for biomaterials.

The packaging industry has experienced rapid expansion in recent decades, intensifying its contribution to the global environmental crisis due to the predominant use of fossil-fuel-derived polymeric materials and the inefficient management of post-consumer waste [22]. More than 400 million tonnes of plastic waste are generated annually, a substantial proportion of which originates from single-use packaging [22]. The non-biodegradable nature of conventional plastics enables their persistence in the environment for centuries, leading to severe accumulation in landfills and marine ecosystems [23]. This situation not only places increasing pressure on waste management systems but also promotes the fragmentation of plastics into micro- and nanoplastics, thereby amplifying ecological and human health risks [24]. These challenges underscore the urgent need for sustainable, biodegradable, and high-performance alternatives for packaging applications.

Therefore, this study aims to evaluate the effect of different HBDs during the DES pretreatment of sulphite pulp, with the purpose of examining the structural and chemical modifications induced in cellulose and assessing its suitability for subsequent functionalization and use as a support material in sustainable packaging technologies. Sulfite cellulose pulp was selected as the substrate due to its high purity, low lignin content, and reproducible composition, which make it suitable for comparative pretreatment studies [19]. According to the literature, its fibrillar structure exhibits high swelling capacity and internal fibrillation, facilitating solvent penetration and increasing the effective reactive surface area [25]. These features may enhance DES–cellulose interactions and enable controlled modification. Furthermore, the preserved cellulose I crystalline structure and moderate degree of polymerization provide a balance between integrity and reactivity, supporting the development of bio-based materials with tunable interfacial and barrier properties [26,27].

Aligned with the principles of green chemistry, this approach significantly reduces the use of conventional reagents through a one-step process, employing DES formulated with carboxylic acids recognized as low-impact and environmentally favourable chemicals. In doing so, the study seeks to generate knowledge applicable to the development of sustainable biomaterials, contributing to the improvement of current cellulose-to-derivatives conversion methods and addressing industrial challenges associated with achieving more efficient, effective, and environmentally responsible production processes.

## 2. Materials and Methods

### 2.1. Materials and Reagents

Sulphite pulp from spruce was supplied by Domsjö (Örnsköldsvik, Sweden). Choline chloride (98%) was obtained from Sigma-Aldrich (Merck Life Science S.L.U., Madrid, Spain), acetic acid (96%) from Panreac (PanReac Química S.L.U., Castellar del Vallès, Spain), citric acid (99%) from Carlo Erba (CARLO ERBA Reagents S.A., Sabadell, Spain), and lactic acid (90%) from Ultimate Fluka (Honeywell, Seelze, Germany).

### 2.2. Deep Eutectic Solvent Preparation and Pretreatment Procedure

#### 2.2.1. Preparation of Acid-Based DESs

DES were prepared by mixing choline chloride (ChCl) with the corresponding hydrogen bond donors (HBDs): lactic acid (LA), acetic acid (AA), and citric acid (CA) at molar ratios of 1:4, 1:8, 1:4, and 1:1 (mol/mol), respectively. The ChCl:LA and ChCl:AA mixtures were stirred at 50 °C for 30 min until a homogeneous, colourless liquid was obtained. In order to reduce the higher viscosity of ChCl:CA mixture (131,000 mPa·s) [28], 30% (*w*/*w*) distilled water was added after mixing the components for 1 h at 80 °C as reported in our previous work [20]. All DESs were stored at 25 °C until use.

#### 2.2.2. Sulphite Pulp Pretreatment Conditions

Pretreatments were performed using 1 g of sulphite pulp at a liquid–solid ratio (LSR) of 25:1 (*v*/*w*) at 110 °C for 4 h in Pyrex bottles placed in a sand bath with orbital shaking (120 rpm). After reaction, the DES was recovered by adding an antisolvent composed of acetone (99.8%) and distilled water (1:1, *v*/*v*), maintaining an LSR of 25:1 (*v*/*w*). The mixture was stirred at 250 rpm for 30 min using an orbital shaker (Optic Ivymen System, Comecta S.A., Madrid, Spain). The pretreated pulp was then washed with distilled water (LSR 50:1, *v*/*w*) and dried at 50 °C for 24 h in an oven (Celsius 2007, Memmert, Schwabach, Germany).

### 2.3. Characterization of Non-Pretreated and DES-Pretreated Sulphite Pulp

#### 2.3.1. Chemical Composition Analysis

Non-pretreated and DES-pretreated pulps, dried to constant weight, were analyzed for polysaccharide and total lignin content following NREL protocols (NREL/TP 510 42618) [29]. Glucose concentration was quantified by HPLC (Agilent 1200, Palo Alto, CA, USA) equipped with a refractive index detector and an Aminex HPX 87H ion exclusion column (Bio Rad Laboratories, Hercules, CA, USA, 300 × 7.8 mm, 9 µm), using calibration standards from 0.1 to 4 mg/mL. Glucan content was calculated according to NREL guidelines [29]. Total lignin was determined as the sum of acid soluble lignin (ASL) and Klason lignin (KL) [29]. ASL was quantified by diluting the hydrolysate with 4% (*w*/*w*) H_2_SO_4_ and measuring absorbance at 205 nm using a UV–Vis spectrophotometer (Libra S60, Biochrom, Cambridge, UK). The solid residue obtained after hydrolysis was dried at 105 °C and considered KL. All analyses were performed in triplicate.

#### 2.3.2. Morphological Characterization

Fibre morphology of untreated and DES-pretreated sulphite pulp was analyzed using a MorFi Compact analyser (Techpap, Grenoble, France) equipped with a CCD camera and MorFi v9.2 software. Four independent measurements were performed per condition, analyzing approximately 120,000 fibres per test at a suspension concentration of 25 mg/L. Parameters quantified included fibre length, fibre width, fines content, and aspect ratio, following [19].

Microstructural features were examined using a TESCAN Clara II ultra-high resolution field emission scanning electron microscope (FESEM, Madrid, Spain). Samples were mounted on 200 mesh copper grids and stained with 1% uranyl acetate. Imaging was performed in STEM mode using a transmitted electron detector. At least three representative micrographs were acquired per sample.

#### 2.3.3. Physicochemical and Crystallinity Analysis

FTIR-ATR spectra of untreated and DES-pretreated pulps were acquired using a Thermo Nicolet 6700 FTIR spectrometer (Thermo Fisher Scientific, Madison, WI, USA) equipped with a Smart Orbit diamond ATR accessory. Spectra were recorded from 4000 to 400 cm^−1^ at 4 cm^−1^ resolution with 20 co-added scans using a DTGS detector with a KBr window.

Crystallinity changes were assessed using the Lateral Order Index (LOI), calculated from the absorbance ratio of characteristic cellulose bands according to Equation (1) [30]; total crystalline index (TCI) was obtained as the quotient between the values of absorbances of bands at 1372 and 2900 cm^−1^ [31] (Equation (2)); and the hydrogen bond intensity (HBI) was quantified as the ratio between 3400 and 1320 cm^−1^ absorbances [32] (Equation (3)):

(1) $$LOI=\frac{A_{{1437\;cm}^{-1}}}{A_{{898\;cm}^{-1}}}$$

(2) $$TCI=\frac{A_{{1372\;cm}^{-1}}}{A_{{2900\;cm}^{-1}}}$$

(3) $$HBI=\frac{A_{{3400cm}^{-1}}}{A_{{1320\;cm}^{-1}}}$$

X ray diffraction (XRD) was performed using a Siemens D500 diffractometer (Siemens AG, Munich, Germany) over a 2θ range of 2–45°, with a step size of 0.02° and a step time of 0.5 s. The Crystallinity Index (*CrI*) was calculated following [20] using Equation (4):

(4) $$Crl=\left[\frac{I_{cry}\;-I_{am}}{I_{cry}}\right]\times 100$$

where *I_cry_* corresponds to the intensity of the crystalline peak at 2θ = 22.8°, and *I_am_* was taken at the minimum of the amorphous valley between the 16° and 22° peaks, which in our diffractograms occurs around 2θ ≈ 19–19.5°.

### 2.4. Enzymatic Pretreatment Analysis

The non-pretreated sulphite pulp and the DES-pretreated samples were subjected to enzymatic hydrolysis using an enzyme loading of 10 FPU per gram of dry substrate (FPU/gds), following previously established methodologies [19]. For this purpose, 0.5 g of sample was suspended in a solid–liquid ratio of 1:30 (*w*/*v*) using 50 mM sodium citrate buffer at pH 4.8, and incubated at 150 rpm for 72 h, with aliquots collected at defined time intervals [20]. At the end of the hydrolysis, the enzymes were denatured in a water bath at 100 °C for 5 min [20]. The released sugars in the aliquots were quantified by high-performance liquid chromatography (HPLC) using an Agilent 1100 system equipped with DAD, FLD, RID, and UV–visible detectors. Chromatographic separation was performed on an Aminex HPX 87H column (Bio Rad Laboratories, Hercules, CA, USA) using 5 mM sulfuric acid as the mobile phase at a flow rate of 0.6 mL min^−1^, with the column temperature maintained at 50 °C. Compound identification and quantification were carried out by comparison with authentic standards analyzed under identical retention time conditions [33].

Enzymatic digestibility was calculated based on the chemical composition results and the glucose released during enzymatic hydrolysis, as quantified by HPLC (Equation (5)) [17].

(5) $$Glucan\;digestibility\;\left(\%\right)=\left[\frac{Glucose\;amount\;in\;enzymatic\;hydrolysate\;\left(g\right)\times 0.9}{Glucan\;amount\;in\;substrate\;\left(g\right)}\right]\times 100$$

### 2.5. Statistical Analysis

Data obtained from the chemical characterization, fibre morphology (MorFi analysis), and glucose released during enzymatic hydrolysis were subjected to one-way ANOVA using the Statgraphics statistical package (Statgraphics Centurion XVI, version 16.1.11). Statistically significant differences among means were determined by the least significant difference (LSD) test at a 95% confidence level.

## 3. Results and Discussion

### 3.1. Effect of Hydrogen Bond Donor Type on DES Pretreatment of Sulphite Pulp

#### 3.1.1. Chemical Analysis

**Figure 1:**
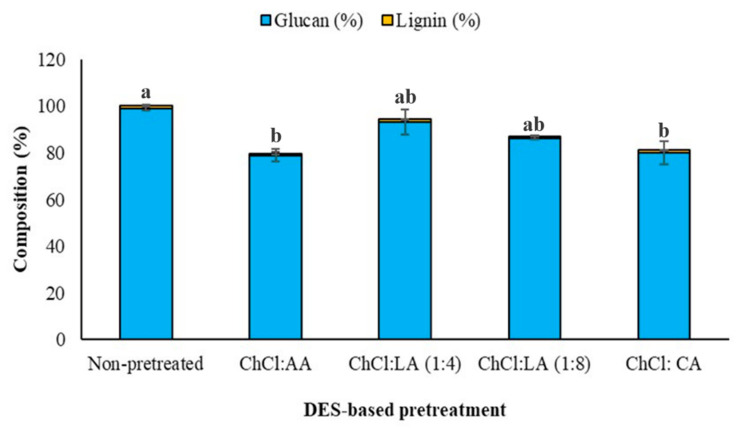
Comparative Effect of DES Pretreatment on the Chemical Composition of Sulphite Pulp.

The chemical composition of the sulphite pulp pretreated with different acidic DESs (Figure 1) showed a significant reduction in cellulose content, reaching approximately 80% for the citric and acetic acid-based systems. This behaviour could be interpreted in light of the “conjoining ion pairs” mechanism described by Li et al. [34], which suggests that cooperative ion-pair formation may contribute to the disruption of the hydrogen-bonding network of cellulose. These ion pairs, generated from the ionized cations and anions of these specific DESs, may facilitate the separation of cellulose chains and prevent their re-aggregation.

These results are consistent with the findings reported by Zhang et al. [35], who demonstrated that the ability of a DES to interact with cellulose depends on the number and strength of hydrogen bonds it can form with the hydroxyl groups of the polymer. This supports the interpretation that DESs with higher acidity, or those with a higher density of carbonyl groups—such as citric and acetic acid—promote stronger interactions with cellulose, thereby enhancing its partial solubilization or component redistribution.

Instead, the lactic acid-based DES produced a moderate reduction in cellulose content. Still, the severity of the pretreatment was a function of the ChCl:LA molar ratio. The ChCl:LA (1:8) system decreased cellulose to approximately 86%, whereas the ChCl:LA (1:4) system resulted in a milder effect, leaving about 93% cellulose in the pretreated sulphite pulp.

These observations are consistent with Zhang et al. [35], who demonstrated that the interaction between the hydrogen bond acceptor (HBA; ChCl) and the hydrogen bond donor (HBD; LA) governs the density of internal hydrogen bonds within the DES. Increasing the proportion of HBD, as in the ChCl:LA (1:8) system, strengthens the internal hydrogen-bonding network, leading to higher viscosity and reduced molecular mobility. Although more viscous DESs diffuse more slowly, they can sustain stronger interactions with cellulose, thereby intensifying pretreatment severity.

Some studies on cellulose dissolution in ChCl-based DESs have shown that the HBA:HBD molar ratio modulates the key physicochemical properties, such as viscosity, ionic mobility, and the availability of chloride anions and HBD molecules to disrupt the intrinsic hydrogen-bonding network of cellulose [20]. In this context, the stronger effect observed for ChCl:LA (1:8) compared with ChCl:LA (1:4) can be attributed to the higher lactic acid content, with higher density of hydrogen-bond donors and carbonyl groups capable of interacting with cellulose, thereby enhancing hydrogen-bond disruption and promoting partial solubilization [36].

Altogether, these findings confirm that both the chemical nature of the HBD and the HBA:HBD molar ratio decisively influence DES pretreatment severity and, consequently, the final cellulose content in the pretreated sulphite pulp.

On the other hand, as shown in Figure 1, the decrease in glucan content after DES pretreatment is accompanied by an expansion of the non-cellulosic fraction, attributable to the partial retention of DES-derived organic acids and lignin–DES associations within the solid phase [37]. This behaviour is consistent with possible hydrogen-bonding interactions between the phenolic and aliphatic –OH groups of lignin and the carbonyl groups (C=O) of the HBD—namely acetic, lactic, or citric acid—as well as with the surface hydroxyl groups of cellulose (O–H···O=C) [38]. In general, lignin phenolic groups act as efficient hydrogen donors, whereas the carbonyl groups of organic acids are excellent hydrogen-bond acceptors. Consequently, DES-derived acids may remain adsorbed onto the residual lignin or cellulose residual even after washing [17]. These mechanisms increase the non-cellulosic mass and generate additional FTIR bands that support this interpretation (provided in Section 3.1.3).

#### 3.1.2. Fibre Morphology and Microstructural Changes

Morphological characterization is essential for evaluating the structural response of sulphite pulp fibres to DES pretreatment. High-resolution automated fibre analysis (MorFi) provides quantitative parameters highly sensitive to chemical-induced alterations in the fibre wall, enabling the detection of fibrillation, delamination, and fragmentation processes that are not evident from bulk chemical composition. Thus, morphological analysis offers a mechanistic link between DES-induced chemical modifications—particularly those driven by acid-based HBDs—and the resulting micro- and meso-scale structural changes in the fibres.

**Figure 2:**
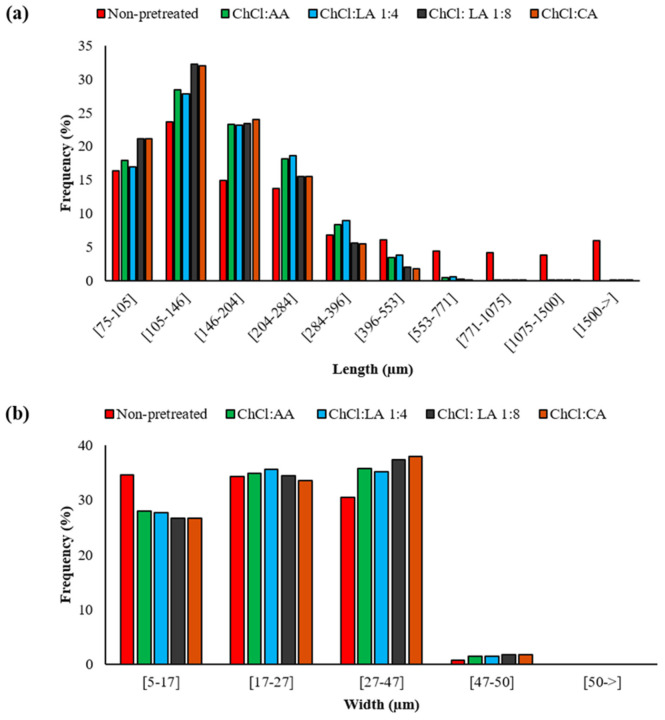
Comparative fibre length (**a**) and fibre width (**b**) distributions for sulphite pulp before and after DES pretreatment.

In this sense, Figure 2a shows the relative frequency of fibres within defined length intervals for the non-pretreated sulphite pulp and the samples pretreated with different acidic DES.

These results indicate that the highest frequency (~32%) in all cases occurs within the intermediate classes (approximately 105–204 µm), although notable differences are observed among treatments. A small proportion of long fibres (553–1500 µm) was detected in the non-pretreated sulphite pulp, but their frequency remained below 5%, indicating that these long fragments constitute only minor components. Overall, the distribution of the non-pretreated pulp was mainly concentrated in the lower and intermediate intervals (105–145 µm), rather than showing a predominance of long fibres, as might be expected.

In contrast, the sulphite pulps pretreated with the different acidic DES exhibited a clear redistribution of fibre lengths. The most acidic DES—particularly ChCl:LA 1:8 and ChCl:CA—generated a higher proportion of short fibres within the 75–145 µm interval, reaching frequencies of approximately 22%, which reflects a more pronounced fibrillation effect. Table 1 summarizes the global averages of fibre length and width for the untreated and DES-treated pulps. The mean fibre length decreases after pretreatment, with ChCl:LA (1:8) and ChCl:CA producing the most pronounced reductions.

According to Mardawati et al. [39], this cell-wall reorganization may arise from the ability of DES to weaken inter- and intramolecular hydrogen bonds, thereby promoting fibrillation and the liberation of fibres that were previously agglomerated rather than inducing destructive fibre cutting, as reported in previous studies. This interpretation is consistent with the redistribution patterns observed in the fibre length profiles (Figure 2a), where DES pretreatment alters the distribution without causing a drastic reduction in the average fibre length.

In contrast, the width distribution of the non-pretreated sulphite pulp and the DES-pretreated samples (Figure 2b) exhibits a similar overall pattern, with all materials—including the untreated pulp—showing the highest frequencies within the narrowest width intervals (approximately 5–47 µm). This indicates that DES pretreatment does not substantially broaden fibre width; instead, it reinforces the predominance of thin fibres, consistent with a fibrillation-driven mechanism.

The reduced frequency in the wider intervals suggests that DES contributes to the disruption of lateral aggregates or the removal of surface-adhered material, without significantly altering the primary structure of the fibre wall. This behaviour supports the interpretation that DES mainly promote fibre dispersion and the separation of bundled structures rather than causing transverse damage.

However, the average values reported in Table 1 reveal a slight increase in fibre width after DES pretreatment, particularly for ChCl:LA (1:8) and ChCl:CA. This apparent widening may be attributed to cell-wall swelling resulting from the weakening of the hydrogen-bonding network and the increased accessibility of water molecules during fibre morphology analysis using the MorFi equipment [40]. The modest increase in the mean fibre width is fully compatible with the distribution patterns observed in Figure 2b and with a more efficient fibrillation process in the most acidic DES. This slight widening is also consistent with previous reports showing that acidic DES can induce cellulose swelling after pretreatment [20]. In lignocellulosic biomass, this effect is typically associated with the partial removal of lignin and hemicellulose, which increases porosity and enzyme accessibility [40,41]. Although sulphite pulp is already highly purified and lacks these heterogeneous components, a comparable swelling phenomenon may still occur through the weakening of the hydrogen-bonding network within cellulose microfibrils [42]. The penetration of DES into the fibrillar matrix can promote internal relaxation and limited expansion of the cell wall, which explains the modest increase in mean fibre width observed after pretreatment [43]. This behaviour may explain the slight increase in the average thickness values of the DES-pretreated samples compared with the non-pretreated pulp.

In this context, the results further indicate that DES pretreatment does not induce severe fibre shortening, particularly when compared with previously reported enzymatic pretreatments [38,44], which are known to generate much more extensive fibre fragmentation. Instead, DES favour fibre individualisation through a combination of swelling, delamination, and controlled fibrillation.

Overall, the combined effects of proton-induced swelling and DES-mediated structural loosening yield individualized fibres with preserved structural integrity, improved dispersion, and a controlled shift toward shorter size classes. These morphological features are advantageous for the development of high-strength polymer-based composites or for reinforcing bioplastics [39].

#### 3.1.3. Chemical Structure Analysis by FTIR-ATR and Crystallinity Index Evaluation

**Figure 3:**
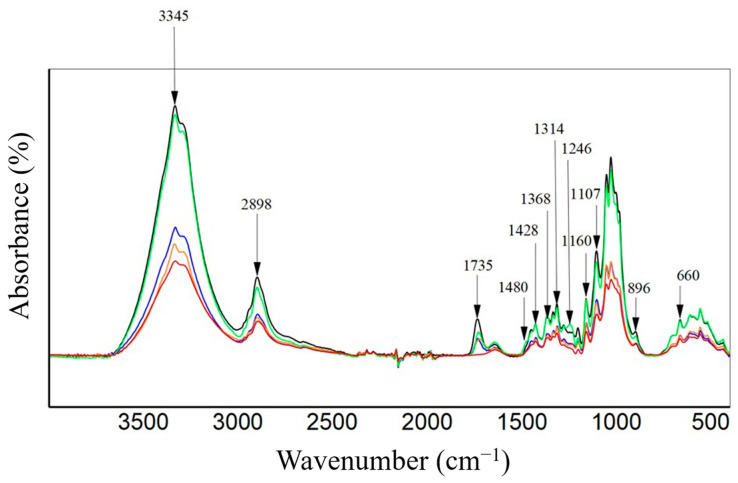
FTIR-ATR spectra of sulphite pulp prior to pretreatment (red) and after DES pretreatment using distinct hydrogen-bond donor systems: acetic acid (green), lactic acid ChCl:LA 1:4 (blue), lactic acid ChCl:LA 1:8 (black), and citric acid (orange).

The FTIR-ATR spectra of non-pretreated and DES-pretreated fibres are presented in Figure 3. The samples pretreated with ChCl:LA (1:8) and ChCl:AA exhibited the most pronounced spectral modifications compared with the non-pretreated sulphite pulp. The broad band at 3345 cm^−1^, assigned to O–H stretching vibrations of cellulose hydroxyl groups, becomes more defined after DES pretreatment, suggesting alterations in the hydrogen-bonding network within the fibrillar structure [45]. Likewise, the band at 2898 cm^−1^, attributed to aliphatic C–H stretching, shows a slight increase in intensity, consistent with the greater exposure of CH and CH_2_ groups following partial disruption of the supramolecular cellulose arrangement [46].

In the region around 1735 cm^−1^, the pretreated sulphite pulp exhibit C=O stretching bands whose origin depends on the HBD used in each DES system. For ChCl:AA corresponds to the C=O stretching of acetyl groups and can be attributed to small amounts of residual acetic acid [17]. In contrast, samples pretreated with ChCl:LA display C=O stretching associated with the carboxyl groups of lactic acid, with the signal being notably more intense in the ChCl:LA (1:8) system [20]. This enhancement suggests a higher proportion of lactic acid interacting with the biomass during pretreatment, possibly leading to partial retention of residual HBD in the pretreated pulp [38].

Similar observations have been reported in previous studies, where FTIR bands at ~1738 cm^−1^ were attributed to slight amounts of residual acetic acid after DES pretreatment. In addition, HPLC analysis of the recycled DES solution confirmed the presence of acetic acid after pretreatment cycles, supporting the plausibility of partial HBD retention under comparable conditions [47]. Nevertheless, this interpretation should be considered a hypothesis supported by indirect evidence, and further confirmatory analyses—such as NMR, TGA-MS, or XPS—would be required to validate this mechanism.

The weak bands observed in the ChCl:CA pretreatment arise from the carboxylic groups of citric acid, although their intensity is typically lower due to the high polarity and limited volatility of this DES system [20].

On the other hand, a small band at 1480 cm^−1^ is observed mainly in the sulphite pulp pretreated with ChCl:AA. This band is associated with C–H deformation modes of the trimethylammonium group of the choline cation present in the DES. In addition, the peak at 1107 cm^−1^ may be related with the C–O stretching vibrations of ChCl [48]. This confirms the presence of small amounts of DES remaining after the pretreatment and washing steps.

Additional structural changes are observed in the fingerprint region. The band at 1428 cm^−1^, associated with symmetric CH_2_ bending, carboxyl group symmetric stretching, and C–H deformation modes, shows a noticeable increase in intensity after pretreatment [32]. The bands at 1368 cm^−1^ and 1314 cm^−1^, corresponding to C–O/C–H stretching and CH_2_ wagging vibrations, respectively, also become more prominent. This collective intensification reflects a greater exposure of these functional groups due to the disruption of densely packed fibrillar regions, leading to increased surface accessibility and partial loosening of the supramolecular cellulose arrangement [49].

The band at 1160 cm^−1^, assigned to C–O–C stretching of cellulose, becomes slightly more defined after DES treatment [32]. Likewise, the increased absorbance at 1028 cm^−1^, corresponding to C–O stretching and C–H in-plane deformation [32], further supports the enhanced exposure of cellulose functional groups following pretreatment.

Finally, the band observed at approximately 660 cm^−1^ can be attributed to vibrational modes associated with O=S=O bonds, confirming the presence of sulfonate groups that may remain linked to the residual lignin, generating lignosulfonate-type structures as a consequence of the sulphite pulping process [19]. In addition, the absorption detected at 1246 cm^−1^ can be assigned to the stretching vibration of sulfonic acid groups (SO_3_H), which are typically reported around 1200 cm^−1^ in protonated lignosulfonates and may shift to higher wavenumbers depending on the degree of protonation and the local chemical environment [50]. In acidic sulphite pulping processes, lignin undergoes extensive sulfonation that remains covalently or ionically associated with the fibre matrix [50]. This behaviour is consistent with the sulfonation mechanism occurring during sulphite pretreatment, in which cleavage of the α-O-4 linkage or removal of the α-hydroxyl group leads to the formation of a resonance-stabilized benzylic carbocation. Subsequently, the HSO_3_^−^ anion adds covalently to the α-carbon, producing β-O-4 units bearing sulfonate groups [51,52]. Therefore, the persistence of these bands after DES pretreatment indicates that these sulphite-derived functionalities are chemically stable under the applied conditions, suggesting that the pretreatment does not significantly disrupt the sulfonate groups originally incorporated into the pulp during the cooking stage.

According to Wichaphian et al. [45], raw cellulose is composed of a combination of crystalline and amorphous regions. The amorphous zones exhibit a lower degree of structural organization and, therefore, a higher susceptibility to degradation, which results in a reduced overall crystallinity. This structural imbalance significantly compromises the mechanical strength, thermal stability, and functional performance of cellulose as a material [53].

The crystallinity index determined using the Segal method [54] is a semi-quantitative parameter based on the difference between the intensity of the crystalline peak and the amorphous valley in the X-ray diffractogram; therefore, it does not represent an absolute value of crystallinity [55]. Consequently, the increase observed in this study should be interpreted as a relative trend toward greater structural order rather than as an absolute quantitative transformation.

In this context, Table 2 presents the values of *CrI*, LOI, TCI, and HBI obtained after pretreatment with the different acidic DES systems. The untreated sulfite pulp exhibited a *CrI* close to 57%, which increased to approximately 70% after the pretreatments, representing a relative increase of ~1.22-fold (~23%). Nevertheless, this increase cannot be attributed solely to the removal of non-cellulosic components. The evidence suggests that the DES systems induce significant rearrangements within the cellulose matrix, as also reported by other authors [56]. This interpretation is consistent with the results of the chemical characterization (Section 3.1.1), where a more pronounced reduction in the cellulosic fraction was observed after pretreatment, indicating the preferential removal of the less ordered regions of cellulose.

The ChCl:AA system showed a notable increase in the parameters associated with structural order (LOI = 1.38; TCI = 0.57; HBI = 3.73), although this effect was not markedly reflected in the *CrI* value (69.4%). This is explained by the fact that *CrI* primarily quantifies the relative proportion between crystalline and amorphous regions, making it less sensitive to internal changes within the crystalline domains [53,54]. In contrast, LOI, TCI, and HBI capture variations in molecular packing and in the intensity of hydrogen-bonding interactions, thereby reflecting more accurately the structural reorganization induced by the DES [57]. The simultaneous increase in these parameters suggests that ChCl:AA promotes the refinement of cellulose I domains, even when the overall crystalline fraction does not vary substantially. This behaviour may be related to the presence of free protons (H^+^) derived from acetic acid, which catalyze the cleavage of ether linkages between residual lignin and the amorphous regions of cellulose [17], facilitating the separation and preferential fractionation of the less ordered domains under moderate conditions [58]. Similar results have been reported for other HBDs, where acetic acid has shown the highest efficiency [17,20], attributed to its smaller molecular size and the stronger electrostatic interaction between the nitrogen cation and its carboxyl group [59].

In the case of the ChCl:LA system, both the 1:4 and 1:8 molar ratios produced no appreciable variations in the structural parameters evaluated. This suggests that, within this range, the proportion of lactic acid does not significantly modify the ability of the DES to interact with the amorphous domains, likely because the accessibility and diffusivity of the HBD within the cell wall remain similar [38]. From an operational perspective, this result is advantageous, as it allows the use of a lower amount of reagent without compromising pretreatment efficiency.

In turn, the ChCl:CA system produced moderate increases in all structural parameters. This behaviour may be attributed to the larger molecular size of citric acid and the presence of three carboxyl groups, which favour the formation of extensive hydrogen-bond networks with ChCl but reduce its diffusivity within the cell wall, thereby limiting its effectiveness [56].

Overall, the results demonstrate that DES pretreatment acts not only through the selective removal of amorphous components but also by promoting the mobility and reorganization of cellulose chains toward more ordered structures. However, the magnitude and nature of these effects strongly depend on the composition of the DES.

#### 3.1.4. Morphological Analysis

**Figure 4:**
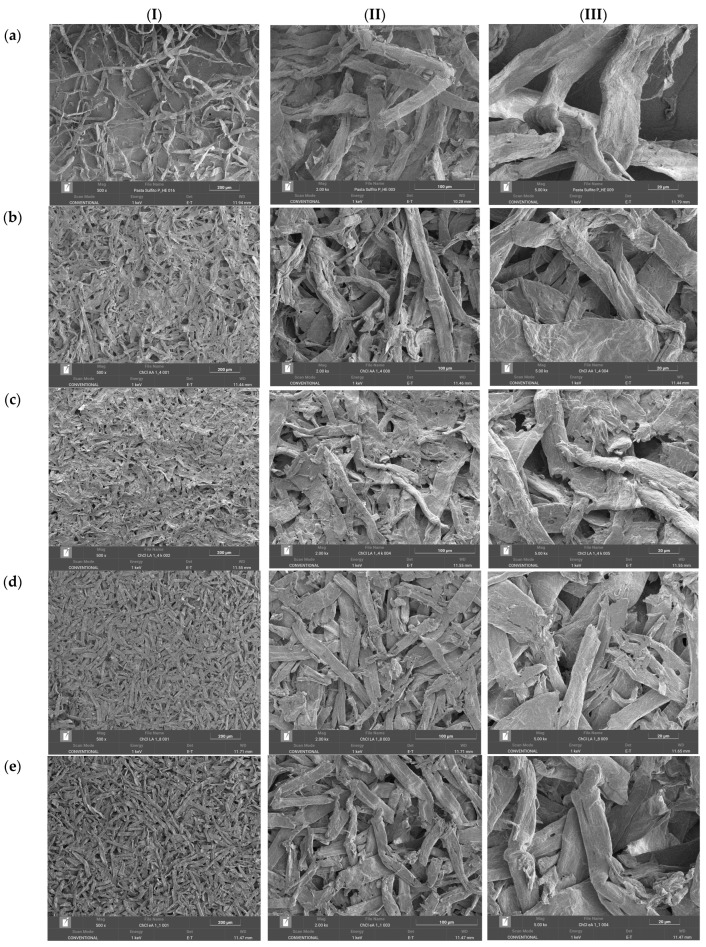
FESEM micrographs of sulphite pulp at different magnifications 200 µm (**I**), 100 µm (**II**), and 20 µm (**III**): (**a**) non-pretreated sample; and samples pretreated with deep eutectic solvents (DES): (**b**) ChCl:AA; (**c**) ChCl:LA (1:4); (**d**) ChCl:LA (1:8); and (**e**) ChCl:CA.

Figure 4 shows the morphological changes in sulphite pulp before and after pretreatment with different deep eutectic solvents (DES). The non-pretreated sample (Figure 4a) exhibits a relatively smooth and compact fibre surface, with limited fibrillation and well-bundled structures. In contrast, all DES-pretreated samples display varying degrees of fibre separation, increased surface roughness, and the appearance of microfibrils, indicating partial disruption of the fibre wall.

The ChCl:AA and ChCl:LA (1:4) treatments (Figure 4b,c) produce moderate fibrillation, with fibres that remain partially compacted but show clear signs of surface swelling and detachment of superficial layers. More pronounced effects are observed for ChCl:LA (1:8) and ChCl:CA (Figure 4d,e), where the fibres appear thinner, more individualized, and less densely packed, forming a more open and porous network. These morphological features suggest stronger cell wall loosening and enhanced delamination when using DES formulations with higher acidity.

According to Lin et al. [41], mild acidic DES pretreatments enhance cellulose reactivity through a combined mechanism of structural loosening and fibre swelling, facilitated by the removal of amorphous components. This process more effectively exposes internal cellulose domains and increases fibrillation, as also observed in the present study (see Figure 4II,III). These findings are consistent with those reported by Tian et al. [40], who evaluated poplar wood pretreated with ChCl:AA to assess its potential for cellulose chemical conversion. In their study, the quantification of the Simon’s staining value (47.6 mg/g) demonstrated that ChCl:AA pretreatment significantly increased the accessible surface area of cellulose and its molecular level porosity, confirming that acidic DES not only remove noncellulosic fractions but also generate a more open and reactive structure. This disruption occurs through competitive hydrogen bonding interactions, in which DES components effectively penetrate the microstructure of cellulose, breaking inter and intramolecular hydrogen bonds between cellulose chains [60]. This, in turn, induces fibre swelling, thereby reducing the energy required for subsequent mechanical defibrillation or even eliminating the need for it [61].

This interpretation is consistent with the results shown in Figure 2, which do not exhibit a drastic decrease in average fibre length but rather a redistribution toward shorter size classes. In addition, the increase in fibre width reported in Table 1 is more plausibly associated with cell wall swelling than with transverse damage induced by pretreatment. Accordingly, the morphological evidence presented in Figure 3 supports the hypothesis that acidic DES do not promote excessive fibre shortening; instead, they disrupt interfibrillar bonding, disaggregate fibre bundles, and release individual fibres that were previously agglomerated.

#### 3.1.5. Enzymatic Hydrolysis Analysis

Since the pretreatment plays a fundamental role in removing amorphous regions of the fibres to isolate crystalline cellulose [62], this study evaluated the enzymatic hydrolysis of non-pretreated and DES-pretreated sulphite pulp with the aim of corroborating and correlating how the increase in crystallinity influences its susceptibility to enzymatic degradation. Recent studies have demonstrated that DES can significantly increase the crystallinity index by removing amorphous fractions and promoting a more ordered and compact structure [45]. Considering that cellulolytic enzymes preferentially act on amorphous domains, a higher crystallinity after pretreatment would imply a reduction in accessibility and, consequently, slower hydrolysis. Under this premise, the comparative analysis between pretreated and non-pretreated samples allows assessing whether the DES-induced increase in crystallinity correlates with a decrease in the rate of enzymatic hydrolysis.

Table 3 presents the kinetics of glucose release during enzymatic hydrolysis. The non-pretreated pulp exhibited the highest glucose release throughout the entire hydrolysis period, reaching approximately 31–32 g/L after 72 h. This behaviour is consistent with the inherently high accessibility of sulphite pulp, whose industrial processing substantially reduces lignin content and facilitates enzymatic attack. This enhanced susceptibility can be attributed to the fact that sulphite pulp is composed predominantly of glucose chains arranged into compact microfibrils, which are tightly packed and aligned to form a dense and highly ordered crystalline structure [63].

In contrast, the DES-pretreated samples showed markedly lower glucose release, suggesting that the structural reorganization induced by the eutectic solvents—particularly the increase in crystallinity—limits enzyme accessibility.

Among the DES systems evaluated, ChCl:LA (1:4) yielded the highest final glucose release (11.80 g·L^−1^), followed by ChCl:LA (1:8) (≈10.8 g·L^−1^). The similarity between both molar ratios indicates that increasing the proportion of lactic acid does not substantially enhance enzymatic susceptibility. This observation aligns with previous reports showing that modifying the HBA:HBD molar ratio in DES does not necessarily improve process performance; instead, such adjustments are often explored to optimize viscosity, acidity, penetration capacity, and overall process cost [64]. From this perspective, using a lower proportion of lactic acid may be advantageous in terms of reagent efficiency and the economic viability of the pretreatment process. Conversely, ChCl:AA and ChCl:CA exhibited the lowest glucose release values (≈6.7 g·L^−1^ and 4.11 g·L^−1^, respectively), consistent with their limited ability to modify the supramolecular structure of cellulose, as previously observed in the FTIR, XRD, and SEM analyses (Section 3.1.1 and Section 3.1.2).

**Figure 5:**
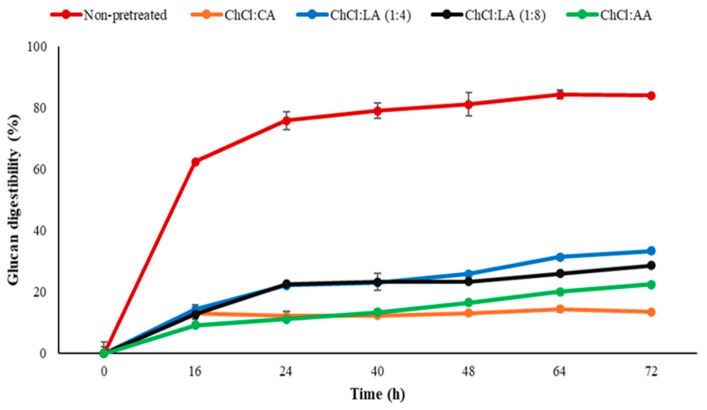
Time-dependent enzymatic digestibility profiles of sulphite pulp subjected to different acidic DES pretreatments.

These findings are further supported by the enzymatic digestibility profiles shown in Figure 5. The non-pretreated pulp reached digestibility values close to 80% at 72 h, whereas the DES-pretreated samples displayed significantly lower digestibility. The lactic-acid-based systems (ChCl:LA 1:4 and 1:8) achieved the highest digestibility among the DES treatments (33.40% and 28.70%, respectively), although still far below the control, while ChCl:AA and ChCl:CA showed the lowest values. The agreement between the glucose-release kinetics (Table 3) and the digestibility profiles (Figure 5) confirms that the DES-induced increase in crystallinity reduces enzymatic accessibility, thereby slowing hydrolysis.

These results are consistent with the XRD and LOI values, indicating an increase in cellulose crystallinity after DES pretreatment. These results could be related to the inability of DES to dissolve crystalline cellulose due to the cohesive enthalpy and the high structural stability of the crystalline domains. Cellulose is stabilized by an extensive network of cooperative hydrogen bonds and strong van der Waals interactions between tightly packed glucan chains, giving rise to a highly ordered, low-energy crystalline network [19]. Breaking this network requires a considerable enthalpic input, which exceeds the interaction energy that the components of DES can provide. Although chloride anions and hydrogen-bond donors in DES can form hydrogen bonds with surface hydroxyl groups, their ability to penetrate the microfibrillar core is limited, as the enthalpic gain derived from DES–cellulose interactions are insufficient to compensate for the enthalpic loss associated with disrupting the crystalline hydrogen-bond network. Consequently, DES preferentially interact with amorphous or surface-exposed regions, while the crystalline microfibrils remain largely intact [65,66].

In contrast, the non-pretreated sulphite pulp exhibits lower TCI and HBI values, which are generally associated with a reduced density of hydrogen-bonding interactions and a partial disruption of the ordered cellulose network [56]. This structural condition enhances the accessibility of amorphous regions to enzymatic attack, as reflected by the higher glucose release and enzymatic digestibility (>80%) observed for the untreated pulp. This consistency is reflected in the higher glucose release observed for the untreated pulp, evidencing its greater enzymatic digestibility. Overall, the lower structural order and weaker hydrogen-bonding interactions observed in the non-pretreated pulp may contribute to improved enzyme adsorption and catalytic activity, which could help explain its comparatively higher hydrolysis performance relative to the DES-treated samples.

Therefore, the results indicate that although acidic DES pretreatments modify the structure of sulphite pulp, these changes do not necessarily lead to improved enzymatic hydrolysis [67]. The combined analysis of XRD, LOI, TCI, and HBI values suggests that DES treatment increases cellulose crystallinity and hydrogen-bonding density, resulting in a more compact and ordered structure. Furthermore, the observed decrease in glucose release and enzymatic digestibility reinforces the notion that the effectiveness of DES pretreatment depends on the type of hydrogen-bond donor and its capacity to modulate the supramolecular organization of cellulose [68].

Although DES pretreatment promotes fibre swelling, fibrillation, and partial delignification—features typically associated with improved accessibility—the enzymatic digestibility results reveal that these morphological changes do not necessarily translate into enhanced hydrolysis. This apparent contradiction can be explained by the coexistence of opposing structural effects: while the pretreatment increases the external surface area and porosity, it simultaneously induces higher crystallinity and possible surface protonation by residual acidic species. These factors may hinder enzyme adsorption and catalytic efficiency, limiting the hydrolysis rate despite the morphological dispersion observed [69,70]. Furthermore, the presence of residual DES components could alter the local microenvironment, affecting enzyme–substrate interactions [17]. This combination of structural and chemical constraints reconciles the observed decrease in enzymatic digestibility with the modifications induced by DES pretreatment.

## 4. Conclusions

This study demonstrates that acid-based deep eutectic solvents offer an effective approach for modifying the chemical and structural properties of sulphite pulp under mild operating conditions, highlighting their potential as a more benign processing medium. Pretreatments using ChCl:AA, ChCl:LA, and ChCl:CA selectively altered glucan content, enhanced the relative structural order of cellulose, and promoted controlled fibrillation without inducing severe fibre shortening. FTIR analysis revealed modifications in hydrogen-bonding interactions and the presence of DES-derived functional groups, while XRD patterns reflected a DES-induced increase in the relative structural order of cellulose.

Morphological analysis showed enhanced fibre individualisation and a visibly more porous cell-wall structure, evidenced by the increased presence of surface cavities following DES pretreatment. Although these structural modifications increased the degree of order within the cellulose matrix, the resulting reorganization of the supramolecular structure led to a reduction in enzymatic digestibility, indicating that DES preferentially interact with amorphous regions while leaving the more ordered, highly crystalline domains largely unaffected.

Altogether, the results highlight the potential of acidic DES as a low-impact system for producing cellulose-based materials with improved physicochemical characteristics.

Notably, the structural features obtained—namely the qualitative increase in structural order, enhanced fibrillation, and controlled chemical modification—may offer advantages for the future development of cellulose-based reinforcement materials intended for biodegradable packaging and bioplastic formulations. By providing an efficient, low-impact pretreatment route that avoids the use of conventional harsh reagents, this work represents a step forward toward the generation of cellulose-derived reinforcement materials through an environmentally benign process, which could contribute to reducing the environmental impact associated with fossil-derived packaging. In this way, the study advances the development of renewable, high-performance materials aligned with the principles of green chemistry and circular technological innovation. Although this study does not evaluate packaging performance, the structural attributes generated through DES pretreatment may be advantageous for subsequent research exploring their incorporation into biodegradable packaging and bioplastic matrices. Further studies assessing mechanical, barrier, and processing properties will be essential to determine their suitability and efficiency in real biopackaging applications.

## Figures and Tables

**Table 1:** Mean arithmetic fibre length and fibre width and standard deviation from non-pretreated and DES-pretreated sulphite pulps.

DES Pretreatment	Mean Fibre Length (µm)	Mean Fibre Width (µm)
Non-pretreated	396 ± 16.28 ^a^	22.10 ± 0.10 ^a^
ChCl:AA	181.75 ± 3.71 ^b^	23.93 ± 0.15 ^b^
ChCl:LA (1:4)	186.50 ± 7.21 ^b^	23.90 ± 0.33 ^b^
ChCl:LA (1:8)	165.25 ± 2.65 ^b^	24.42 ± 0.35 ^b^
ChCl:CA	164.00 ± 0.40 ^b^	24.57 ± 0.04 ^b^

Different letters within the same column show statistically significant differences.

**Table 2:** Crystallinity Parameters (*CrI*, LOI, TCI, and HBI) of Sulphite Pulp Before and After Pretreatment with Acidic DES.

	Crystallinity			
DES Pretreatment	*CrI* (%)	LOI	TCI	HBI
Non-pretreated	57.3	0.99	0.50	3.41
ChCl:AA	69.4	1.38	0.57	3.73
ChCl:LA (1:4)	70.7	1.24	0.50	3.02
ChCl:LA (1:8)	69.4	1.11	0.50	3.60
ChCl:CA	70.3	1.04	0.49	3.64

**Table 3:** Kinetics of glucose release during the enzymatic hydrolysis of non-pretreated and DES-pretreated sulphite pulp.

Glucose Released (g·L^−1^)							
Time (h)	0	16	24	40	48	64	72
Non-pretreated	0.00 ± 0.00 ^a^	23.50 ± 2.60 ^a^	28.62 ± 0.44 ^a^	29.81 ± 0.60 ^a^	30.62 ± 0.42 ^a^	31.80 ± 1.31 ^a^	31.68 ± 0.10 ^a^
ChCl:AA	0.00 ± 0.00 ^a^	2.72 ± 0.19 ^b^	3.33 ± 0.10 ^b^	3.40 ± 0.11 ^b^	4.92 ± 0.11 ^b^	6.00 ± 0.11 ^b^	6.68 ± 0.11 ^b^
ChCl:LA (1:4)	0.00 ± 0.00 ^a^	5.04 ± 0.23 ^b^	7.84 ± 0.30 ^c^	8.18 ± 0.06 ^c^	9.14 ± 0.10 ^c^	11.09 ± 0.08 ^c^	11.79 ± 0.20 ^c^
ChCl:LA (1:8)	0.00 ± 0.00 ^a^	4.80 ± 0.21 ^b^	7.62 ± 0.84 ^c^	8.50 ± 0.22 ^c^	8.77 ± 0.79 ^c^	9.80 ± 0.40 ^c^	10.78 ± 0.62 ^c^
ChCl:CA	0.00 ± 0.00 ^a^	3.93 ± 0.70 ^b^	3.20 ± 0.90 ^b^	3.76 ± 0.46 ^b^	3.97 ± 0.03 ^b^	4.39 ± 0.20 ^b^	4.11 ± 0.28 ^d^

Values represent mean ± SD (n = 3); Different letters within the same column show statistically significant differences.

## Data Availability

All data supporting the findings of this study are included within the article.

## Abbreviations

The following abbreviations are used in this manuscript:

AA	Acetic acid
ASL	Acid-soluble lignin
CA	Citric acid
ChCl	Choline chloride
*CrI*	Crystallinity Index
DAD	Diode Array Detector
FLD	Fluorescence Detector
RID	Refractive Index Detector
DES	Deep eutectic solvents
FPU/gds	Filter paper units per gram of dry substrate
HBA	Hydrogen bond acceptor
HBD	Hydrogen bond donor
HPLC	High-performance liquid chromatography
KL	Klason lignin
LA	Lactic acid
LOI	Lateral Order Index
LSR	Liquid–solid ratio
XRD	X-ray Diffraction
